# Kernel Transcriptome Profiles of Susceptible Wheat Genotypes in Response to Wheat Dwarf Bunt

**DOI:** 10.3390/ijms242417281

**Published:** 2023-12-08

**Authors:** Shenqiang Su, Zihao Zhang, Tong Shen, Jing Chen, Qi Liu

**Affiliations:** 1 Key Laboratory of the Pest Monitoring and Safety Control of Crops and Forests of the Xinjiang Uygur Autonomous Region, College of Agronomy, Xinjiang Agricultural University, Urumqi 830052, China; 15283026603@163.com (S.S.); a2900926255@163.com (Z.Z.); 18331519385@163.com (T.S.); chenj@xjau.edu.cn (J.C.); 2 Key Laboratory of Prevention and Control of Invasive Alien Species in Agriculture & Forestry of the North-Western Desert Oasis, Ministry of Agriculture and Rural Affairs, Urumqi 830052, China; 3 College of Plant Protection, Nanjing Agricultural University, Nanjing 210095, China

**Keywords:** wheat dwarf bunt, *Tilletia controversa* Kühn, transcriptome, RNA sequencing, abnormal kernel

## Abstract

Wheat dwarf bunt is caused by *Tilletia controversa* J. G. *Kühn* (TCK), which is a serious fungal diseases affecting kernels of wheat. In order to identify candidate genes involved in the abnormal development of kernels in wheat, we used RNA sequencing technology to analyze the transcriptome of the abnormal and healthy kernels of a susceptible variety (Yili053) at the mid-filling stage, late-filling stage, and maturity stage, respectively. The differentially expressed genes (DEGs) were analyzed, and there were 3930 DEGs, 28,422 DEGs, and 20,874 DEGs found at the mid-filling stage, late-filling stage, and maturity stage in Yili053, respectively. A total of 1592 DEGs (506 DEGs up-regulated) showed continuously differential expression in the three stages. Gene ontology analysis showed that these DEGs were related to biological regulation, metabolic processes, and the response to stimulus. Kyoto Encyclopedia of Genes and Genomes enrichment analysis showed that these DEGs play major roles in pathways including photosynthesis, carbon metabolism, carbon fixation in photosynthetic organisms, and glyoxylate and dicarboxylate metabolism. Moreover, we predicted that 13 MADS-MIKC transcription factors, which were continuously up-regulated, were crucial for regulating the maturation and senescence of eukaryotes. Some 21 genes related to the plant hormone signaling transduction pathway and 61 genes related to the response to stimulus were analyzed. A total of 26 of them were successful validated with a qPCR analysis. These genes were thought to be involved in the abnormal development of kernels infected by TCK. A transcriptomics analysis of wheat kernels in response to TCK will contribute to understanding the interaction of TCK and wheat, and may provide a basis for knowledge of molecular events in the abnormal development of kernels, which will be helpful for more efficient TCK management.

## 1. Introduction

Wheat (*Triticum aestivum* L.) is one of the most crucial crops grown for basic foods [1,2]. Wheat dwarf bunt (WDB) is caused by *Tilletia controversa* Kühn (TCK), and can reduce yield and quality by infecting wheat kernels [3,4]; total loss might exceed 70–80% [5,6,7]. WDB is a significant global fungal disease, and it has been documented in 15 countries [3,8,9,10,11]. The export of TCK-contaminated wheat from these nations has been subject to restrictions due to the possibility that TCK will spread through spores of the pathogen.

WDB is a seed- and soil-borne disease. Inreases in warfing and tillering are WDB’s characteristic symptoms [12]. Wheat heads are replaced by brown-black teliospores, and sori are formed, also called bunt balls [13]. Usually, all florets are replaced with teliospores in a single spikelet, and the diseased kernels are nearly spherical, rigid, and blocky after being crushed [12]. The teliospores have a rotting fish odor because of the trimethylamine. Even though the rate of infection in the wheat is relatively low, the flour still has distinct smells.

When the pathogen infects the plant, the plant will produce a series of defense responses in order to resist the invasion of the pathogen, such as modification of plant cell walls [14], release of reactive oxygen species [15], production of secondary metabolites [16], and production of pathogenesis-related proteins [17]. These defense responses are associated with disease-causing genes and transcription factors [18]. With the advancement of molecular biological technology, various omics, such as transcriptomics, are widely used in the study of plant–pathogen interaction and gene function.

Using RNA sequencing (RNA-seq) technology to extract total RNA from a given tissue sample and sequence it to identify gene expression levels, it was possible to swiftly and completely gather all the transcriptional data. RNA-seq has proven to be a very effective method of researching the transcriptome expression variations of genes associated with biotic and abiotic stress in wheat. Liu et al. [19] analyzed and compared the gene expression profiling of CMPG1-V transgenic plants and their receptor Yangmai 158 after *Blumeria graminis* inoculation at four infection stages using RNA-Seq. Jitendra et al. [20] compared the transcriptome in two wheat genotypes with contrasting levels of drought tolerance, and found that regulatory genes such as MT, FT, AP2, etc. were involved in defense response in wheat. The comparison of RNA-seq profiles between glumes of wheat groups differing in glumes toughness and rachis brittleness revealed a few DEGs that may be involved in glumes’ toughness and nutrient remobilization [21]. RNA-seq has been used in wheat kernel development. Liu et al. found that the NAC transcription factor NAC019-A1 was a negative regulator of starch synthesis in wheat developing endosperm [22]. Wei et al. performed isoform sequencing for wheat grain and RNA-seq for the embryo and de-embryonated kernels to analyze their transcriptome characteristics and homoeolog expression bias [23]. Consequently, RNA-seq could offer new information about wheat kernel development after TCK infection.

Although extensive research has analyzed wheat–TCK interactions, but there is little research about the molecular mechanism of wheat kernel abnormality caused by TCK; instead, the majority of studies so far have concentrated on TCK detection and risk analysis [24,25]. Therefore, it is crucial to investigate the genes involved in the aberrant growth of kernels after TCK infection. In this study, RNA-seq was used to examine the traits of the healthy and pathological kernel expression in sensitive wheat cultivars (Yili053). The differentially expressed genes of wheat kernels’ development during the mid-filling stage, late-filling stage, and maturity stages were analyzed, exploring the genes related to the abnormal development of kernels after the effect of TCK. This study can be used as a starting point for further research into the molecular mechanisms underlying the emergence of abnormal kernels in TCK-affected wheat, which would help in managing and preventing the disease.

## 2. Results

### 2.1. RNA Sequencing Analysis

In this study, the 18 cDNA libraries (9 TCK-infected and 9 control) were created, containing about 9.76 Gb clean reads of each sample (Table 1). The average GC contents were 54.51% and 57.81% for the control and infected samples, respectively. Q30 values were up to 93.42% and 93.87% for control and infected samples, indicating that a high-quality library was generated.

### 2.2. DEGs Identification

**Figure 1:**
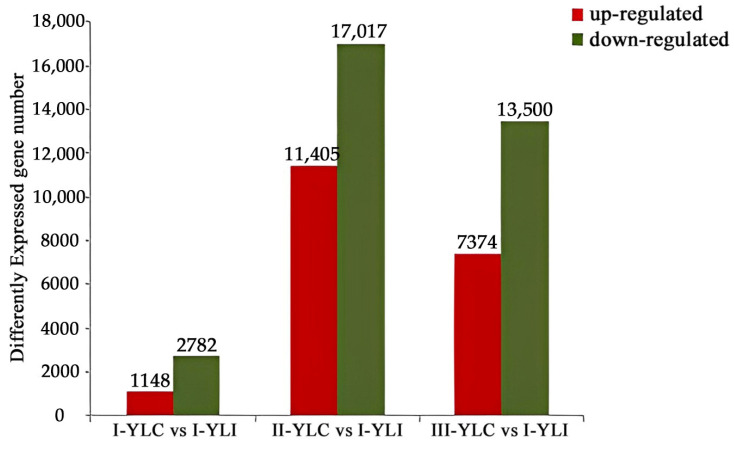
The overall distribution of DEGs in TCK-infected vs. control samples at three growth periods. Note: Comparison group: I-YLC and I-YLI in mid-filling stage; II-YLC and II-YLI in late-filling stage; III-YLC and III-YLI in maturity stage.

The differentially expressed genes were recognized by the number of reads, with FPKM used to normalize. As a result, there were 3930, 28,422 and 20,874 DEGs found in three periods (Figure 1). The most DEGs were found at the late-filling stage, with 11,405 up-regulated genes and 17,017 down-regulated genes. After the infection of TCK, the number of DEGs increased at late-filling stage and then decreased at the maturity stages.

### 2.3. Gene Ontology Analysis of DEGs

**Figure 2:**
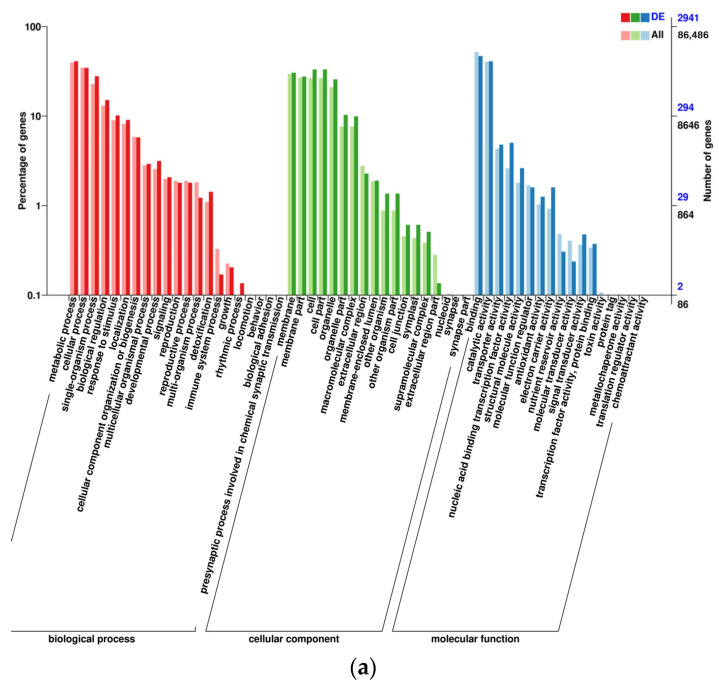

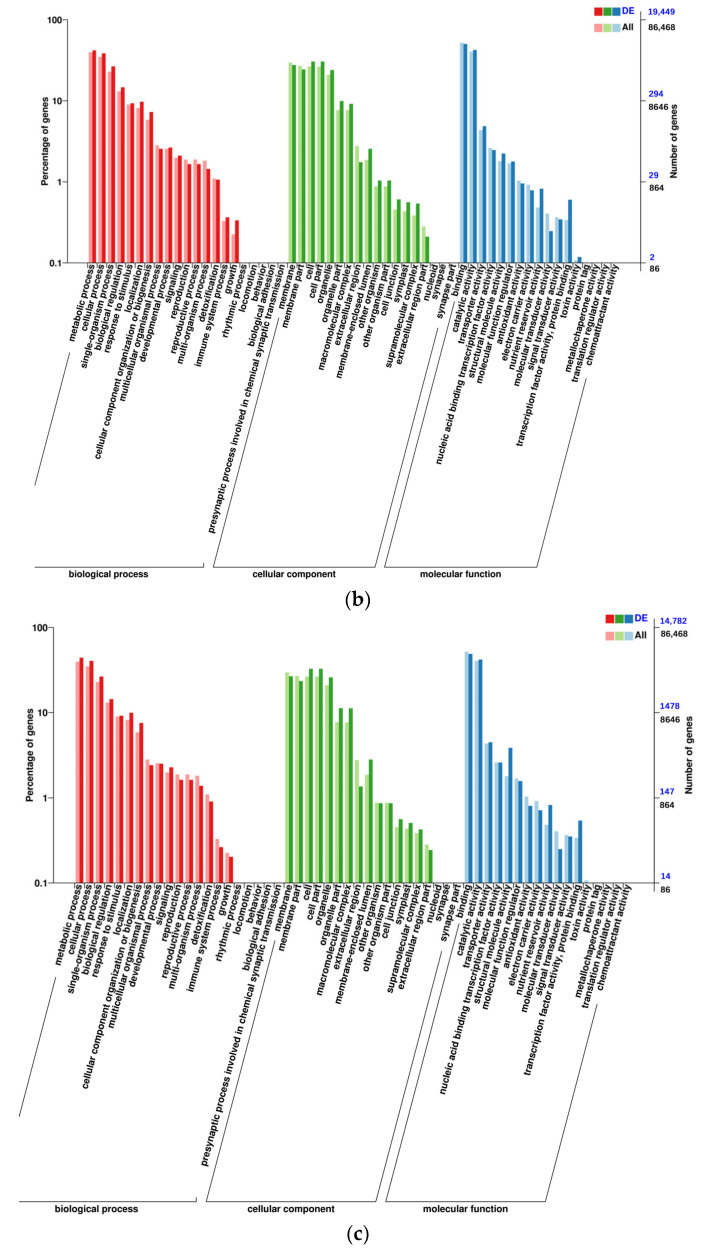
GO analysis of DEGs. Note: (**a**) Comparison group: YLC and YLI in mid-filling stage; (**b**) YLC and YLI in late-filling stage; (**c**) YLC and YLI in maturity stage. X-axis: Go terms and classifications; Y-axis: Number of DEGs (genes) annotated to the term (**right**) and percentage of that in all DEGs (genes) (**left**).

The Gene ontology (GO) analysis was conducted to assess the function of DEGs, which focused primarily on biological process, cellular component, and molecular function (Figure 2). These DEGs were notably enriched 56 GO terms. In the category of biological process, DEGs related to functions such as metabolic processes, cellular processes, single-organism processes, biological regulation, response to stimulus are abundant. It had been proposed that the response of wheat to TCK involved a significant number of genes associated with cell metabolism and stress response. More DEGs had been annotated in the category of cellular component, demonstrating that the TCK infection affects the components of wheat kernel cells and might be connected to the aberrant growth of wheat grains. The development of wheat kernels and the catalytic reaction of enzymes were also impacted by the abundance of genes associated with binding function and catalytic activity in the category of molecular function. Figure 2 showed the GO enrichment in DEGs in all genes, which indicated the importance of a specific GO term in DEGs and all genes, respectively. The terms with two bars significantly different from each other can be picked up as potential targets for further analysis on functions, since these GO terms were enriched differently between DEGs-based and all-gene-based enrichment.

### 2.4. Cluster of Orthologous Groups Analysis of DEGs

**Figure 3:**
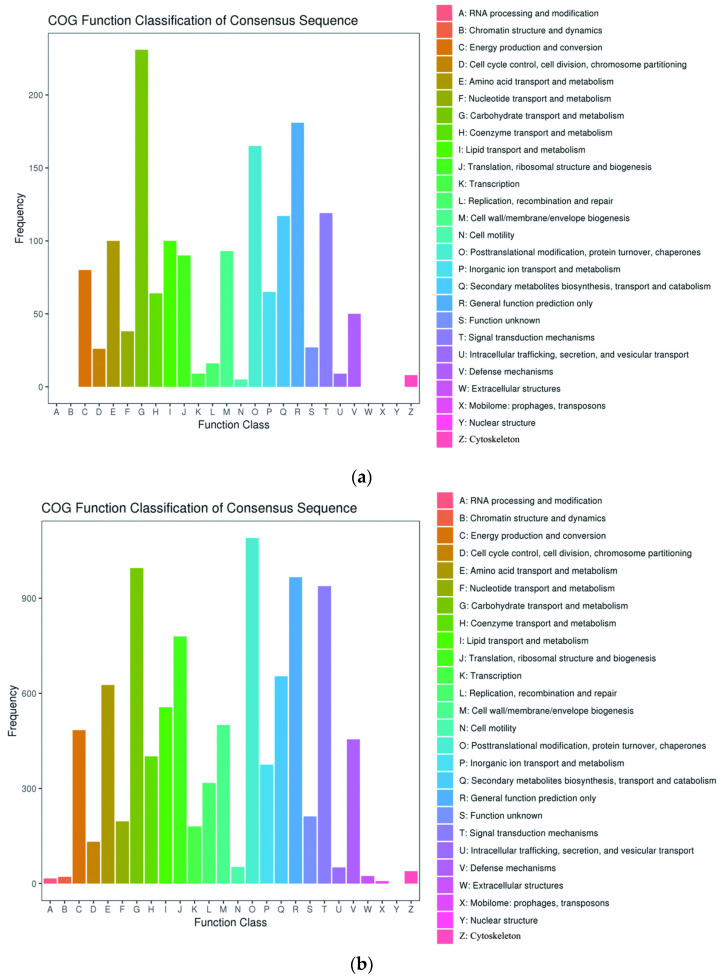

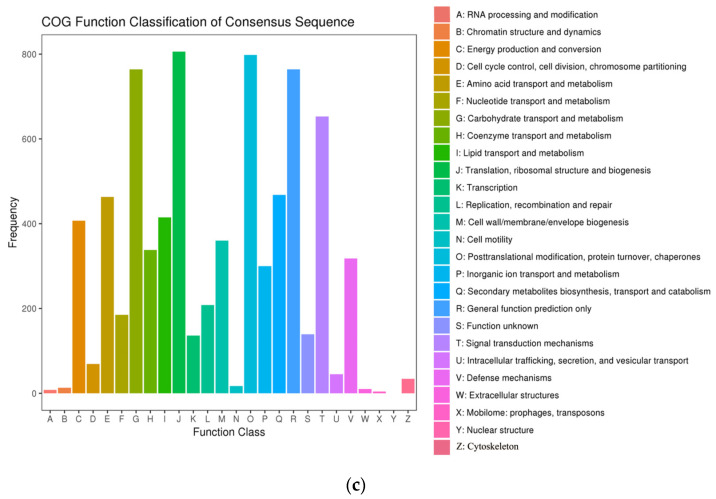
Comparison of COG databases. Note: (**a**) Comparison group: YLC and YLI in mid-filling stage; (**b**) YLC and YLI in late-filling stage; (**c**) YLC and YLI in maturity stage.

Clusters of Orthologous Groups (COG) analysis can analyze the evolution of DEGs and predict the function of proteins. These DEGs were compared to the database and divided into 26 functional categories (Figure 3). At the mid-filling stage, the gene functions were involved in carbohydrate transport, metabolism, post-translation modification, protein turnover, chaperones and general function prediction. In addition to the aforementioned three functions, translation, ribosomal structure, biogenesis, and signal transduction mechanisms were involved in at the late-filling stage and maturity stage.

### 2.5. KEGG Pathway Enrichment Analysis of DEGs

**Figure 4:**
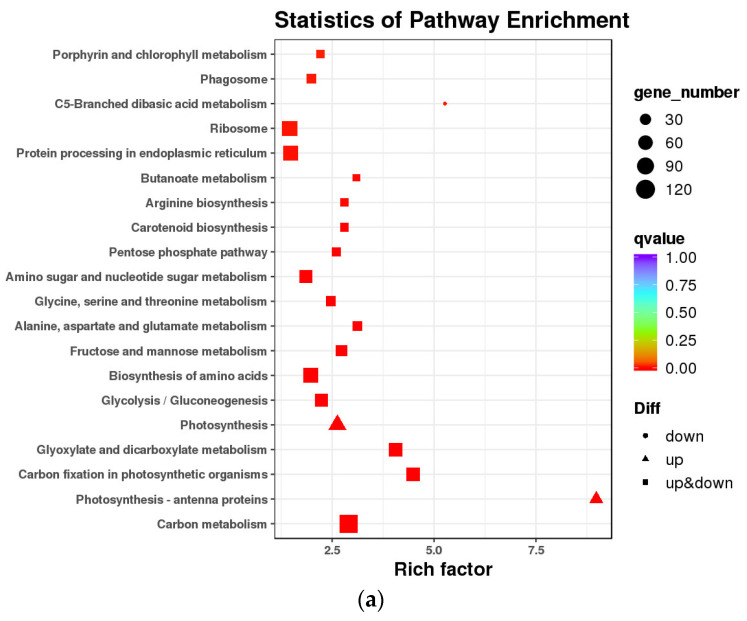

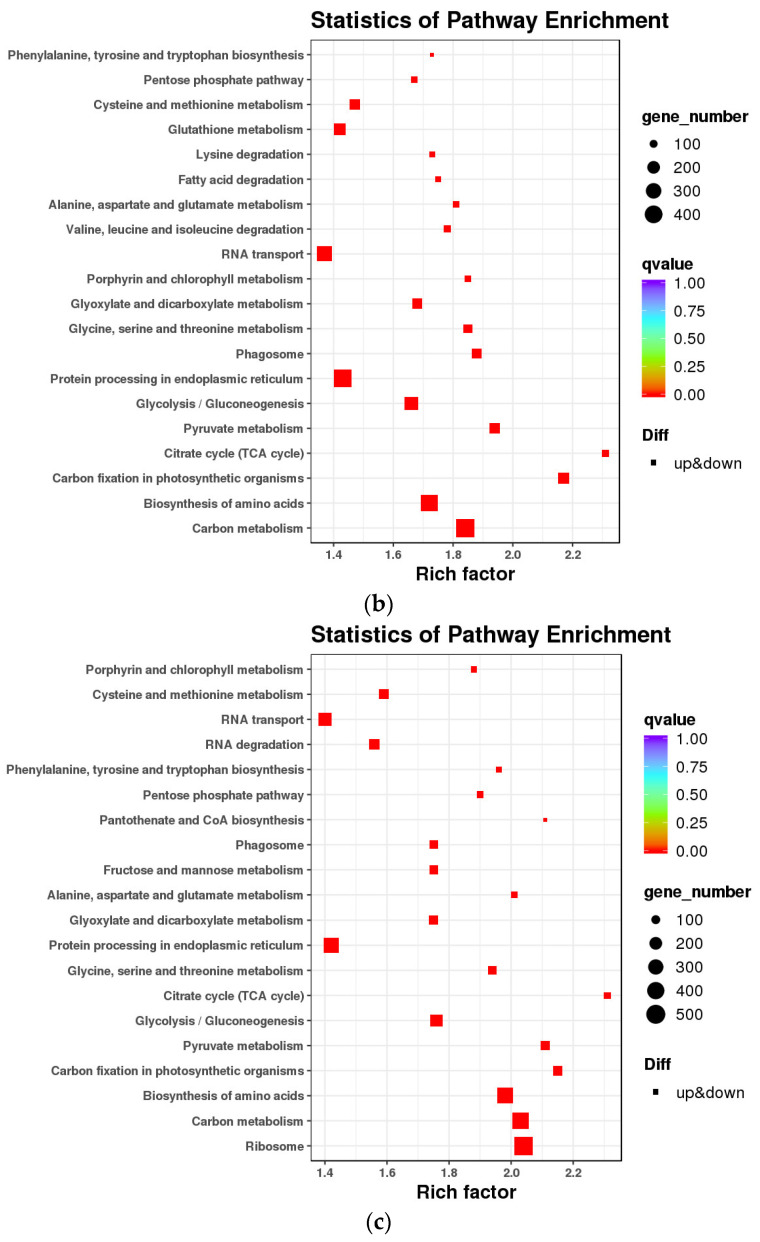
Enrichment scatter map of KEGG pathway. Note: (**a**) Comparison group: YLC and YLI in mid-filling stage; (**b**) YLC and YLI in late-filling stage; (**c**) YLC and YLI in maturity stage.

Kyoto Encyclopedia of Genes and Genomes (KEGG) was used to examine the DEGs participating in signal transduction and metabolism pathways. The top 20 pathways that were considerably enriched and had the lowest q-value were chosen after the DEGs and KEGG databases were compared (Figure 4).

Carbon metabolism had the highest gene enrichment (121DEGs) and photosynthesis-antenna proteins had the highest enrichment factor value (8.98) at the mid-filing stage. Only up regulated genes were enriched in photosynthetic antenna proteins and photosynthesis pathways, while only down regulated genes were enriched in the C5-Branched dibasic acid metabolism pathway. During the late-filing stage, carbon metabolism had the largest gene enrichment (480 DEGs), the endoplasmic reticulum’s protein processing had the second-highest enrichment factor value (442 DEGs), tricarboxylic acid cycle (TCA cycle) had the highest enrichment factor value (2.31). During the mature stage, ribosome (525 DEGs) and carbon metabolism (420 DEGs) were the two most strongly enriched metabolic pathway, the TCA cycle being the most highly enriched factor value (2.31).

### 2.6. Candidate Genes Related to Abnormal Kernels Development

**Figure 5:**
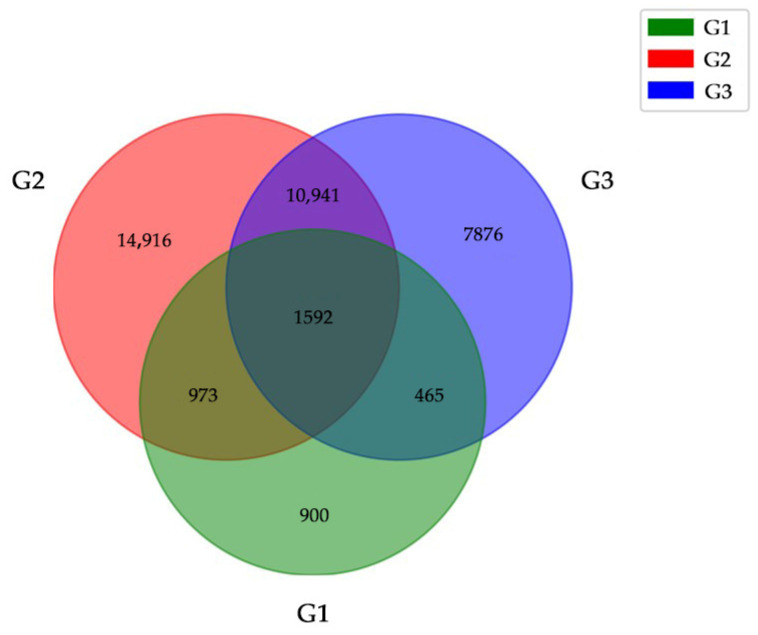
Venn diagram analysis of the DEGs in Yili 053. G1 represents the shared DEGs of I-YLC vs. I-YLI; G2 represents the shared DEGs of II-YLC vs. II-YLI; and G3 represents the shared DEGs of III-YLC vs. III-YLI.

**Figure 6:**
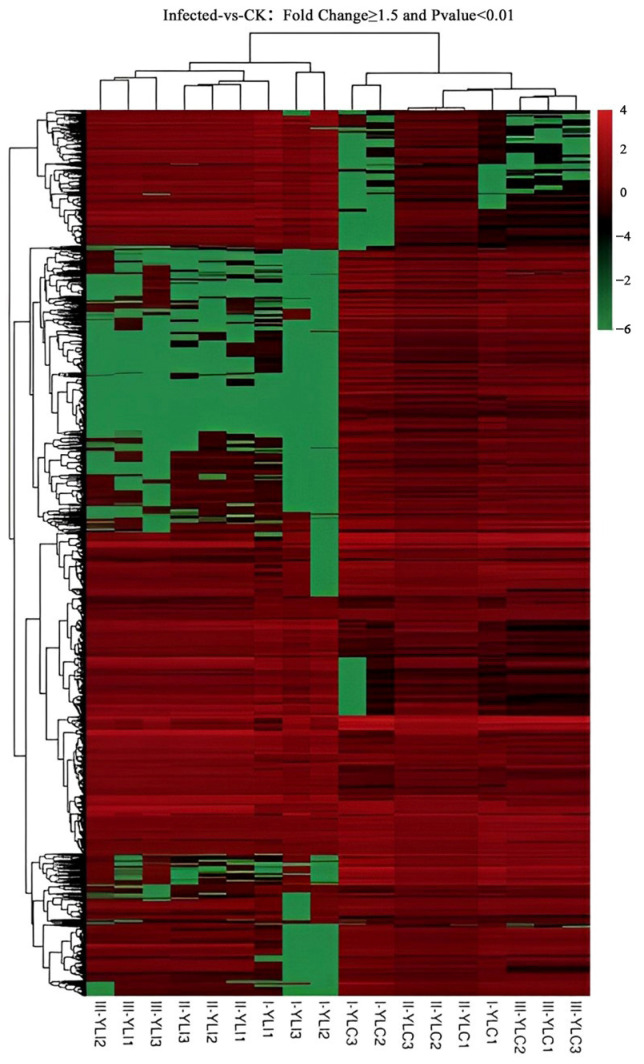
Heatmap of Yili 053 continuously expressed DEGs.

**Figure 7:**
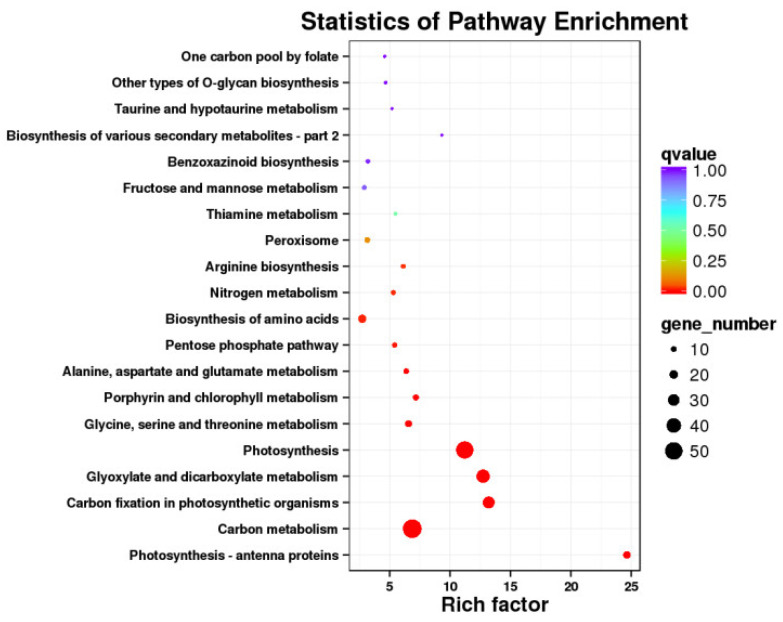
Enrichment scatter map of KEGG pathway.

The shared DEGs in infected and control samples were analyzed using venn diagram, which continuously differentially expressed in three growth stages of wheat, were selected as candidate genes. A total of 1592 DEGs were screened that were shown in Figure 5. Heatmap of constantly expressed DEGs was displayed in Figure 6, 506 genes were shown to be consistently up-regulated. The KEGG annotation showed that the 506 DEGs play major roles in pathways including photosynthesis, carbon metabolism, carbon fixation in photosynthetic organisms, and glyoxylate and dicarboxylate metabolism (Figure 7).

### 2.7. Transcription Factors Prediction

**Figure 8:**
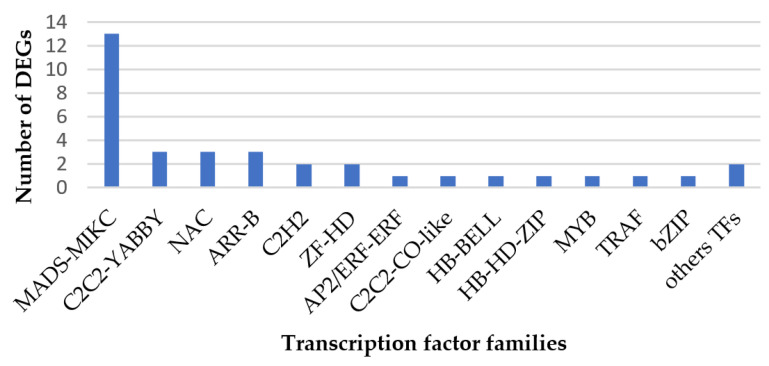
DEGs of transcription factors, which were continuously up-regulated.

Transcription factors (TFs) are key components involved in the transcriptional regulatory system. A total of 5050 TFs were identified in three growth periods; among them, 127 TFs were identified to be consistently differentially expressed, 35 of which were shown to be continuously up-regulated during the three growth periods. These TFs included 13 MADS-MIKC, 3 C2C2-YABBY, 3 NAC, 3 ARR-B, 2 C2H2, 2 ZF-HD, 1 AP2/ERF-ERF, 1 C2C2-CO-like, 1 HB-BELL, 1 HB-HD-ZIP, 1 MYB, 1 TRAF, 1 bZIP, and 2 other TFs (Figure 8).

### 2.8. DEGs Related to Plant Hormone Biosynthesis

DEGs related to the plant hormones of infected kernels were analyzed. A total of 32 genes were continuously differentially expressed in three stages (Table 2). Most of these DEGs were down-regulated in infected kernels; only three DEGs were continuously up-regulated. These DEGs were enriched in the pathway of plant hormone signal transduction, the MAPK signaling pathway, protein processing in the endoplasmic reticulum, and glutathione metabolism.

### 2.9. DEGs Involved in Response to Stimulus

DEGs related to response to stimulus in infected kernels were identified. There were 61 genes that showed continuously differentially expression in the three stages. Following the annotation of KEGG, the DEGs were enriched in nine pathways, including photosynthesis–antenna proteins, protein processing in the endoplasmic reticulum, glycerophospholipid metabolism, plant hormone signal transduction, butanoate metabolism, galactose metabolism, glutathione metabolism, the MAPK signaling pathway, and alpha-linolenic acid metabolism. During the three stages, there were 19 genes that showed continuous upregulation. Most of these genes were enriched in the photosynthesis–antenna protein metabolic pathway (Table 3).

### 2.10. qRT-PCR Analysis

**Figure 9:**
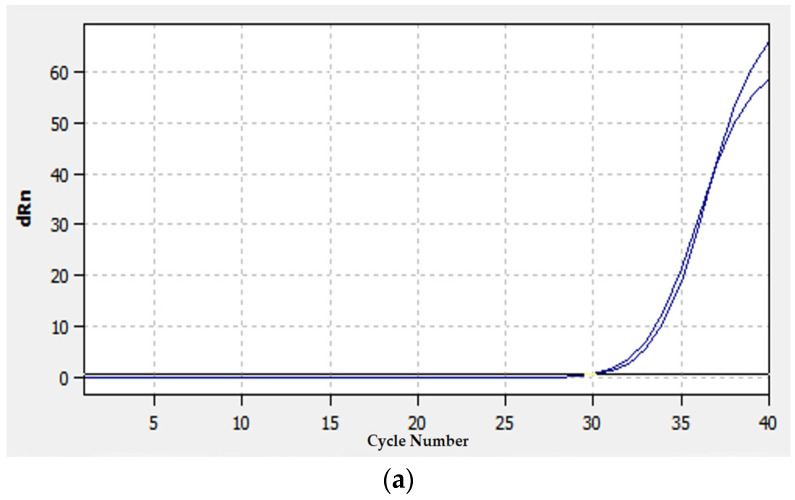

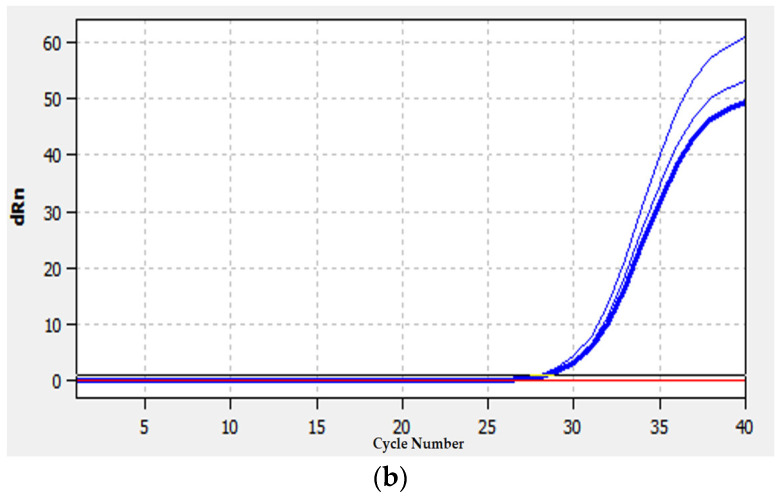
Amplification curve of DEGs that were continuously up-regulated. Note: (**a**) *TraesCS4B02G122300* gene; (**b**) *TraesCS7D02G419400* gene. Blue line represents the sample signal, the red line represents the baseline.

The expression level of 24 randomly selected DEGs was detected by qRT-PCR. The expression results were consistent with the results of RNA-Seq analysis (Table 4, Figure 9). This indicated that the results of RNA sequencing were reliable.

## 3. Discussion

The occurrence of and change in biological processes were closely related to the regulation of gene expression and the transcriptome. Proteins encoded by mRNA play a major role in life activities. These mRNAs are the bond that connects genes to proteins, and are the most important form of regulation. In order to clarify the molecular mechanism of the abnormal development of kernels in wheat infected by TCK, RNA-Seq was used to analyze the transcription level of three growth stages of the wheat cultivar Yili053. The results suggested that the TCK infection could cause changes in the transcriptome in wheat, that is, these DEGs might be related to the abnormal development of kernels.

There were 3930, 28,422, and 20,874 DEGs in Yili 053 during the mid-filling stage, the late-filling stage, and maturity stage, respectively. The late-filling stage had the highest number of DEGs. Therefore, it was concluded that the late-filling stage was the key stage at which wheat kernels respond to infection by TCK. The results of GO annotation showed that a lot of DEGs were enriched in biological regulation, metabolic processes, and the response to stimulus. The GO results support that the infection of pathogen could change the primary (plant growth) and secondary (defensive reaction) metabolism of plants. This indicated that energy is expended via the metabolism in relation to plant defense [26,27,28,29]. Ren et al. [30] clarified that a large number of genes related to cellular and metabolic processes are differentially expressed in wheat after infection by TCK. The results of this study were consistent with those of previous studies.

KEGG analysis showed that most DEGs in three growth stages of wheat were enriched in photosynthesis, carbon metabolism, and carbon fixation in photosynthetic organisms. Photosynthesis of leaves mainly serves to provide photosynthates for plant vegetative organ building, but only some of the photosynthates are transported to the developing grain [31]. After flowering, wheat leaves gradually age, and photosynthetic function decreases. At this time, non-leaf photosynthetic organs such as the spike, peduncle, and sheath of wheat can still proceed with photosynthesis, and provide carbon assimilates for wheat grain filling [32]. Our results showed that the wheat may achieve grain filling by enhancing grain photosynthesis after TCK infection.

Transcription factors play an important role in regulating plant growth and development. Our studies showed that 13 MADS-MIKC transcription factors were continuously up-regulated, and played an important role in regulating the maturation and senescence of eukaryotes. MADS-box transcription factors could affect the integrity of cell walls by regulating the expression of PKC genes in the phosphatidylinositol signaling system [33]. NAC transcription factors play an important role in almost every stage of growth and stress conditions in plants [34,35]. However, in our study, only two NAC transcription factors were continuously up-regulated; the reasons for this will be studied in the future. ZF-HD family proteins were widely distributed in terrestrial plants [36]. Many studies have confirmed that plant ZF-HD transcription factors are involved in many biological processes, such as growth, abiotic stress, and plant hormone response [37]. In our study, it was found that ZF-HD transcription factor could also respond to biotic stresses such as TCK. Some 35 transcription factors were identified continuously up-regulated in infected kernels, as found using RNA-Seq. The differential expression of these transcription factors indicated that they could respond to TCK infection.

The plant hormone signal transduction pathway played an important role in the development of wheat kernels; the formation of many kernels is affected through the corresponding signal transduction pathway. Some studies have shown that polyamines, as an important plant growth regulator, have a significant effect on the grain filling of cereal crops. Spraying exogenous polyamines could significantly promote grain filling and the weight of kernels in wheat [38]. A higher ratio of abscisic acid (ABA) was beneficial to the development of endosperm cell and grain filling, which increased the rate of grain filling and the weight of kernels [39]. As a plant growth regulator, ABA can regulate the translocation of photosynthetic products to kernels, and promote the synthesis of starch [40]. At the same time, heteroauxin also promotes the accumulation of dry matter in kernels [41]. Many studies have shown that plant hormones can regulate wheat grain filling and promote the accumulation of starch, which is of great significance in the development of wheat kernels. In this study, most of the genes involved in the plant hormone signal transduction pathway showed a pattern of down-regulation, indicating that infection by TCK inhibited the plant hormone signal transduction pathway; this might be an important reason for the abnormal development of wheat kernels. The inhibition of plant hormone signal transduction leads to the failure of grain filling, and infection by TCK in wheat kernels tissue resulted in the formation of sori.

## 4. Materials and Methods

### 4.1. Fungal Material and Culture

TCK was provided by the United States Department of Agriculture, Agricultural Research Service, Aberdeen, ID, USA. The 2% soil–agar medium plates containing TCK teliospores were cultured in an incubator (MLR-352H-PC, Panasonic, Osaka, Japan) at 5 °C for 24 h light cycle. Under an automated inverted fluorescence microscope (IX83, Olympus, Tokyo, Japan), teliospore germination and hyphal development could be seen after being parafilm-covered for 60 days. The hyphae were collected, combined with distilled water, and used to inoculate wheat at a concentration of 10^6^ spores per milliliter with an OD_600_ of 0.15 [18].

### 4.2. Plants Material and Inoculation

Wheat cultivar (Yili053) was provided by the Institute of Crop Science, Chinese Academy of Agricultural Sciences, Beijing, China, and was susceptible to TCK. The protocol in this study complied with relevant institutional, national, and international guidelines and legislation.

Wheat seeds were sterilized for 1 min with 30% NaClO and then rinsed three times with sterile water. Seeds were vernalized for a month at 5 °C in plates. Seedlings were transferred into pots with soil and organic matter in a 1:2 ratio after being vernalized, then transferred to growth chambers (ARC-36, Percival, Perry, IA, USA). Ten seedlings were planted in each pot, and six pots were utilized for inoculation, while six pots were used as controls. Wheat seedlings were grown in conditions with 14 h light/10 h dark cycle at 5 °C during the tillering stage. Additionally, wheat seedlings were raised at 25 °C throughout the boot stage. The wheat spikes were injected with 1 mL teliospore suspensions during the early boot stage, while the spikes were still covered by the leaf sheaths. The inoculation procedure was performed three times with a one-day interval [18]. Meanwhile, the control treatment samples received the same amount of sterile water, and were raised under identical circumstances.

### 4.3. RNA Extraction, cDNA Library Construction, and Sequencing

Kernels from three distinct heads of the wheats that had been inoculated and the controls were collected at the mid-filling stage, late-filling stage, and maturity stages, respectively. Each treatment contained three replications. Kernel samples were promptly flash-frozen in liquid nitrogen and stored at −80 °C. Total RNA was extracted from the infected and control samples at specific periods using the TRNzol Universal Reagent Kit (Tiangen, Beijing, China), following the manufacturer’s instructions. The purity and concentration of the RNA were detected using NanoDrop 2000 (Thermo Fisher Scientific, Wilmington, DE, USA). RNA integrity was assessed using the RNA Nano 6000 Assay Kit (Agilent Technologies, Santa Clara, CA, USA). mRNA was enriched using poly-T oligo-attached magnetic beads, followed by the enzymatic fragmentation; first-strand cDNA was synthesized using a random hexamer primer and M-MuLV reverse transcriptase, while the second strand was synthesized using RNase H and a DNA polymerase I system. The ds-cDNA samples were then purified using an AMPure XP system (Beckman Coulter, Beverly, MA, USA) followed by A-tailing, adapter ligation, and then enrichment by PCR. The PCR products were purified with an AMPure XP system, and the quality of the library was assessed with an Agilent Bioanalyzer 2100 system [30]. Sequencing was carried out with an Illumina HiSeq2500 system at Biomarker Technologies Co. Ltd. (Beijing, China).

### 4.4. Quality Control, Mapping, and DEGs Screening

After removing adaptor sequences, low-quality reads, and reads containing ploy-N, the raw data were converted into clean reads, and Q30 and GC content were computed. The Hisat2 (v2.0.4) tool [42] was used to map the clean reads to the IWGSC RefSeq 1.1 reference genome with default configuration. Cufflinks (version 2.2.1) was used to measure FPKM (reads per kilobase of exon model per million mapped reads) values [43], then the DEGs between infected and control samples in three periods were assessed with DESeq2 [44] R package (v1.6.3). An adjusted *p*-value < 0.01 and log_2_FC (fold change) > 1.5 were chosen as the thresholds for significantly different expression. Candidate genes related to abnormal kernel development were those in which differential expression was consistently observed within the three periods between infected and control kernels.

### 4.5. Functional Annotation of DEGs

Gene ontology [45] was implemented with the GOseq R package (v3.10.1) based on Wallenius non-central hyper-geometric distribution [46], which can adjust for gene length bias in DEGs. *p*-values < 0.05 were considered significant GO terms. Clusters of orthologous group terms (COG) [47] and Kyoto Encyclopedia of Genes and Genomes (KEGG) [48] enrichment analyses were carried out to predict possible functional classifications and molecular pathways, respectively. COG is a protein database generated by comparing the protein sequences of complete genomes. Each cluster contains proteins or groups of paralogs from at least three lineages. We used an eggnog-mapper to predict functions. KEGG [49] is a database resource for understanding the high-level functions and utilities of biological systems, such as the cell, the organism and the ecosystem, from molecular level information, especially large-scale molecular datasets generated by genome sequencing and other high-throughput experimental technologies (http://www.genome.jp/kegg/ (accessed on 10 March 2023)). We used KOBAS (v3.0) [50] software to count enrichment of DEGs in KEGG pathways.

### 4.6. Validation of RNA-Seq Results Using qRT-PCR

The expression profiles of randomly selected DEGs at different growth stages were analyzed using qRT-PCR. The GAPDH gene was used as an endogenous control [51]. Table 5 contains a list of the primers used in this experiment. Three replicates were employed for each gene. qTOWER 2.0/2.2 quantitative real-time PCR thermal cyclers (Jena, Germany) were used to conduct qRT-PCR. Reverse transcription was performed using a TUREscript first-stand cDNA SYNTHESIS Kit (AiDLAB Biotech, Beijing, China) according to the manufacturer’s instructions. SYBR^®^ Green (Thermo Fisher) was used as a detection dye. All genes underwent pre-denaturation for 3 min at 95 °C, followed by 40 cycles of 95 °C for 10 s (denaturation), 60 °C for 30 s (extension), and 72 °C for 30 s in a 20 uL reaction volume. The specificity of the amplification was verified by a melt curve analysis (from 60 to 95 °C). The 2^−ΔΔCt^ method was used to determine each gene’s expression level [52].

## 5. Conclusions

In this study, the transcriptome characteristics of infected and healthy kernels at three time points were analyzed. As far as we know, this is the first manuscript that describes the abnormal development of wheat kernels affected by TCK at the molecular level. There were 3930 DEGs selected at the mid-filling stage, 28,422 DEGs at the late-filling stage, and 20,874 genes at the maturity stage. Additionally, we predicted 13 MADS-MIKC transcription factors, which were crucial for regulating the maturation and senescence of eukaryotes. We have found 21 genes related to the plant hormone signaling transduction pathway, and 61 genes related to the response to stimulus. A total of 26 of them were successfully validated by our qPCR analysis. These identified putative target genes may be related to the abnormal development of kernels infected by TCK. These results will aid in our understanding of TCK’s infection mechanism, which will provide a theoretical framework for its scientific management.

## Figures and Tables

**Table 1:** Summary statistics of RNA-Seq data.

Numbers	Samples	Clean Reads	Clean Bases	GC Content	Q30 (%)
1	I-YLC1	33,846,470	10,118,821,246	55.69%	95.46%
2	I-YLC2	38,588,627	11,517,825,572	54.84%	95.00%
3	I-YLC3	37,958,897	11,343,101,536	54.37%	95.01%
4	I-YLI1	37,731,986	11,276,627,552	57.82%	93.91%
5	I-YLI2	35,408,404	10,561,674,364	58.70%	93.97%
6	I-YLI3	43,665,455	13,042,812,834	58.55%	93.90%
7	II-YLC1	37,424,109	11,172,643,120	51.80%	91.54%
8	II-YLC2	39,668,019	11,817,094,732	52.53%	91.17%
9	II-YLC3	43,770,044	13,052,803,514	51.85%	91.92%
10	II-YLI1	41,044,118	12,272,430,466	56.42%	93.91%
11	II-YLI2	41,626,954	12,447,001,058	56.32%	93.99%
12	II-YLI3	43,680,494	13,053,542,618	56.40%	93.96%
13	III-YLC1	41,024,047	12,249,838,096	57.04%	93.24%
14	III-YLC2	42,236,376	12,634,731,488	56.04%	94.14%
15	III-YLC3	36,526,906	10,892,077,864	56.40%	93.28%
16	III-YLI1	43,832,299	13,101,853,390	58.51%	93.54%
17	III-YLI2	43,363,058	12,976,495,304	58.83%	93.85%
18	III-YLI3	45,101,790	13,488,891,936	58.73%	93.80%

Note: I-YLC represents control samples in the mid-filling stage, II-YLC represents control samples in the late-filling stage, III-YLC represents control samples in the mature stage. I-YLI represents infected samples in the mid-filling stage, II-YLI represents infected samples in the late-filling stage, III-YLI represents infected samples in the maturity stage.

**Table 2:** DEGs related to plant hormones.

Gene ID	I-YLC vs. I-YLI	II-YLC vs. II-YLI	III-YLC vs. III-YLI	KEGG_Pathway_Annotation
*TraesCS4D02G258000*	up	up	up	-
*TraesCS5B02G162100*	up	up	up	-
*TraesCS7D02G419400*	up	up	up	Plant hormone signal transduction (ko04075)
*TraesCS1B02G229400*	down	down	down	MAPK signaling pathway—plant (ko04016); Plant hormone signal transduction (ko04075)
*TraesCS1B02G237400*	down	down	down	-
*TraesCS1D02G146900*	down	down	down	Plant hormone signal transduction (ko04075)
*TraesCS1D02G218200*	down	down	down	MAPK signaling pathway—plant (ko04016); Plant hormone signal transduction (ko04075)
*TraesCS2A02G309300*	down	down	down	Plant hormone signal transduction (ko04075)
*TraesCS2D02G234100*	up	down	down	Protein processing in the endoplasmic reticulum (ko04141)
*TraesCS3A02G006600*	down	down	down	-
*TraesCS3A02G145300*	down	down	down	Plant hormone signal transduction (ko04075)
*TraesCS3A02G233000*	down	down	down	Plant hormone signal transduction (ko04075)
*TraesCS3A02G307100*	down	down	down	Plant hormone signal transduction (ko04075)
*TraesCS3A02G371800*	down	down	down	Plant hormone signal transduction (ko04075)
*TraesCS3A02G372000*	down	down	down	Plant hormone signal transduction (ko04075)
*TraesCS3A02G372100*	down	down	down	Plant hormone signal transduction (ko04075)
*TraesCS3B02G007400*	down	down	down	-
*TraesCS3B02G404300*	down	down	down	Plant hormone signal transduction (ko04075)
*TraesCS3B02G404400*	down	down	down	Plant hormone signal transduction (ko04075)
*TraesCS3D02G292100*	down	down	down	Plant hormone signal transduction (ko04075)
*TraesCS3D02G364900*	down	down	down	Plant hormone signal transduction (ko04075)
*TraesCS4B02G161800*	down	down	down	Plant hormone signal transduction (ko04075)
*TraesCS4D02G210900*	down	up	up	MAPK signaling pathway—plant (ko04016); Plant hormone signal transduction (ko04075)
*TraesCS4D02G231900*	down	down	down	-
*TraesCS5A02G243900*	down	down	down	-
*TraesCS5A02G265600*	down	down	down	Plant hormone signal transduction (ko04075)
*TraesCS5B02G265300*	down	down	down	Plant hormone signal transduction (ko04075)
*TraesCS5D02G206700*	down	down	down	MAPK signaling pathway—plant (ko04016); Plant hormone signal transduction (ko04075)
*TraesCS5D02G432700*	down	up	up	Glutathione metabolism (ko00480)
*TraesCS7B02G019600*	down	up	up	-
*Triticum_aestivum_newGene_3982*	down	down	down	Plant hormone signal transduction (ko04075)
*Triticum_aestivum_newGene_8549*	down	down	down	-

**Table 3:** DEGs related to response to stimulus.

Gene ID	KEGG_Pathway_Annotation
*TraesCS1A02G403300*	Photosynthesis–antenna proteins (ko00196)
*TraesCS1B02G432700*	Photosynthesis–antenna proteins (ko00196)
*TraesCS1D02G411300*	Photosynthesis–antenna proteins (ko00196)
*TraesCS2A02G206200*	Photosynthesis–antenna proteins (ko00196)
*TraesCS2D02G209900*	Photosynthesis–antenna proteins (ko00196)
*TraesCS3B02G133400*	Galactose metabolism (ko00052)
*TraesCS4B02G122300*	-
*TraesCS4B02G200100*	Glutathione metabolism (ko00480)
*TraesCS4D02G258000*	-
*TraesCS4D02G319100*	-
*TraesCS5A02G322500*	Photosynthesis–antenna proteins (ko00196)
*TraesCS5A02G350600*	Photosynthesis–antenna proteins (ko00196)
*TraesCS5B02G162100*	-
*TraesCS5B02G572400*	Glutathione metabolism (ko00480)
*TraesCS5D02G413300*	Alpha-linolenic acid metabolism (ko00592)
*TraesCS6A02G372100*	-
*TraesCS6D02G152700*	Photosynthesis–antenna proteins (ko00196)
*TraesCS6D02G356100*	-
*TraesCS7A02G177700*	Protein processing in the endoplasmic reticulum (ko04141)

**Table 4:** Validation of RNA sequencing by qRT-PCR.

Gene ID	qRT-PCR	FPKM	Validated	Gene ID	qRT-PCR	FPKM	Validated
*TraesCS1A02G403300*	0.2 ± 0.10 gh	44.83	+	*TraesCS5B02G572400*	0.64 ± 0.03 ef	30.59	+
*TraesCS1B02G432700*	0.28 ± 0.01 gh	55.71	+	*TraesCS5D02G413300*	2.9 ± 1.67 a	8.4	+
*TraesCS2A02G206200*	0.32 ± 0.13 gh	32.49	+	*TraesCS6D02G152700*	1.65 ± 0.15 c	24.14	+
*TraesCS2D02G209900*	0.37 ± 0.05 fg	37.23	+	*TraesCS7A02G177700*	0.95 ± 0.63 d	24.43	+
*TraesCS3B02G133400*	0.03 ± 0.11 h	76.43	+	*TraesCS7D02G419400*	0.22 ± 0.03 gh	12.13	+
*TraesCS4B02G122300*	0.19 ± 0.29 gh	4.81	+	*TraesCS1B02G229400*	0.26 ± 0.2 gh	5.12	+
*TraesCS4B02G200100*	0.30 ± 0.11 gh	24.14	+	*TraesCS2A02G309300*	0.12 ± 0.06 gh	4.34	+
*TraesCS4D02G258000*	0.13 ± 0.01 gh	56.0	+	*TraesCS2D02G234100*	0.16 ± 0.09 gh	5.29	+
*TraesCS4D02G319100*	1.97 ± 0.72 b	4.02	+	*TraesCS3D02G364900*	0.17 ± 0.1 gh	19.24	+
*TraesCS5A02G322500*	0.43 ± 0.41 fg	15.25	+	*TraesCS4D02G210900*	0.3 ± 0.27 gh	15.05	+
*TraesCS5A02G350600*	1.48 ± 0.02 c	63.58	+	*TraesCS5D02G206700*	0.32 ± 0.27 gh	3.49	+
*TraesCS5B02G162100*	0.76 ± 0.13 de	38.2	+	*TraesCS5D02G432700*	0.4 ± 0.96 gh	214.13	+

Note: a–h represents significant difference in the amount of gene expression detected by qRT-PCR. “+” represents the gene that can be detected by qRT-PCR.

**Table 5:** Primers of selected DEGs for expression analysis using qRT-PCR.

Gene ID	Forward Primer (5′-3′)	Reverse Primer (5′-3′)
*TraesCS1A02G403300*	ATGTTCGGCTTCTTCGTG	CCAGGCGTTGTTGTTGAC
*TraesCS1B02G432700*	TTCTCCATGTTCGGCTTCT	CCAGGCGTTGTTGTTGAC
*TraesCS2A02G206200*	CTGGTGATCGGGTACATC	CAGAGTCTCCTTCTTCTCC
*TraesCS2D02G209900*	ATCGGGTACATCGAGTTC	CAGAGTCTCCTTCTTCTCC
*TraesCS3B02G133400*	GCTACAACACCGAGAATG	CTAGACCCGAATCTCCAA
*TraesCS4B02G122300*	CCTGAAGCTCTCCTACAC	GCTGGTCGTAGTAGAGTG
*TraesCS4B02G200100*	TGCTGCCTGATGATTCTG	TCGCTGAACTTTCCCAAG
*TraesCS4D02G258000*	GTGGTGCTCTACGACCTC	TAGGCGTCCAGGTTGTTG
*TraesCS4D02G319100*	TGGCTCTTCAGTTCCTCT	GACCTTCTTCTTCTCCTTGG
*TraesCS5A02G322500*	TTCTCCATGTTCGGCTTCT	CCAGGCGTTGTTGTTGAC
*TraesCS5A02G350600*	ATGTTCGGCTTCTTCGTG	CCAGGCGTTGTTGTTGAC
*TraesCS5B02G162100*	AGCGGCATATTTACTTCG	TCAGCATCAAGGTAGGTT
*TraesCS5B02G572400*	CCGAGAAGTTGCTGTCAC	CTGAGGTCTGCGATGGAT
*TraesCS5D02G413300*	GATCTTGCTGCCAATGCT	AAGTTGATGCGGTCCTTG
*TraesCS6D02G152700*	AAGAGCGAGAAGGAGATG	TGAGGATGTTGTTGTTGAC
*TraesCS7A02G177700*	AGCTTCTTCTCGCAGGAC	CCTCCATCAGAGACAGCAG
*TraesCS7D02G419400*	CCAGTCCAAGAACCAGTA	CGACGACAGGTAGAAAGT
*TraesCS1B02G229400*	CATACGATTCAAGGAGGTT	GAAAGTAGTTGCTGGAAGA
*TraesCS2A02G309300*	CTAACTGTCTCAGGTGATG	CTTCTAACTTGGCGACTT
*TraesCS2D02G234100*	GGCAATAGACTCTGTTCAGG	GGCTCCATCAGGTTCTTC
*TraesCS3D02G364900*	CGGCAGTCTTCCATCTTC	ACTCCTCGGCATTCCATA
*TraesCS4D02G210900*	ATACGGCGGCATAGACTG	TACTTGGCGGGCTCAATC
*TraesCS5D02G206700*	CGTTCATTGGCTGCTTCTA	GGTCAAGATTGCCGACTC
*TraesCS5D02G432700*	CTGGACATCCTCAAGACC	CTCTCGTAGCTGTGGAAC

## Data Availability

Data are contained within the article.
